# Delayed reconstruction is associated with higher rates of medial meniscus and chondral injury following anterior cruciate ligament (ACL) injury: A New Zealand ACL registry study

**DOI:** 10.1002/ksa.70002

**Published:** 2025-08-18

**Authors:** Richard Rahardja, Hamish Love, Mark G. Clatworthy, Simon W. Young

**Affiliations:** ^1^ Department of Surgery, Faculty of Medical and Health Sciences University of Auckland Auckland New Zealand; ^2^ Axis Sports Medicine Christchurch New Zealand; ^3^ Auckland Bone & Joint Surgery Auckland New Zealand; ^4^ Department of Orthopaedic Surgery North Shore Hospital Auckland New Zealand

**Keywords:** ACL reconstruction, anterior cruciate ligament, anterolateral ligament, lateral extra‐articular tenodesis

## Abstract

**Purpose:**

Early reconstruction for anterior cruciate ligament (ACL) rupture may be controversial, with some clinicians opting for a trial of non‐operative management first. The impact of delayed surgery on outcomes is unclear, but it may be associated with an increase in secondary intra‐articular pathology involving the menisci and cartilage. This study aimed to analyze the association between the timing of surgery and outcomes, including revision ACL reconstruction, concomitant meniscal and chondral injuries.

**Methods:**

Prospective data recorded in the New Zealand ACL Registry were analyzed. Primary ACL reconstructions performed between April 2014 and December 2022 were included. Timing of surgery was categorized into five groups: ≤6 weeks, 6 weeks to 3 months, 3–6 months, 6–12 months and >12 months. Revision rates and incidence of concomitant meniscal and chondral injury were compared between the five groups of surgical timing.

**Results:**

A total of 15,586 primary ACL reconstructions were analyzed, of which 1263 were performed within 6 weeks (8%), 3718 between 6 weeks to 3 months (24%), 5129 between 3 and 6 months (33%), 3223 between 6 and 12 months (21%) and 2253 more than 12 months (14%). The incidence of medial meniscal tears was greatest when surgery was delayed 6–12 months (40%, adjusted odds ratio [aOR] = 1.1, *p* = 0.01) and more than 12 months after injury (53%, aOR = 2.0, *p* < 0.001). Delayed surgery more than 3 months was associated with an increasing incidence of chondral injury (aOR > 1.3, *p* < 0.001). Revision rates were lowest in patients who underwent delayed surgery more than 12 months after injury (adjusted hazard ratio [HR] = 0.6, *p* < 0.001), but differences in activity levels, age, sex and graft choice were noted.

**Conclusion:**

Delayed ACL reconstruction is associated with a greater incidence of concomitant medial meniscal and chondral injury and should be considered when trialling non‐operative management for ACL rupture.

**Level of Evidence:**

Level II.

**Abbreviations**
ACL: anterior cruciate ligament
CI: confidence interval
HR: hazard ratio
IQR: interquartile range
NR: not recorded
n.s.: not significant
OR: odds ratio
PROM: patient‐reported outcome measure
SD: standard deviation

## INTRODUCTION

The optimal timing of when to perform anterior cruciate ligament (ACL) reconstruction following rupture is unclear [1, 15, 35, 47, 48, 54]. Some clinicians prefer a trial of non‐operative management first, followed by delayed reconstruction [19, 21, 46, 49]. However, it is unclear what impact delaying surgery may have on patient outcomes. Previous studies have reported that the timing of surgery may influence clinical outcomes, including re‐rupture, secondary intra‐articular pathology and patient‐reported outcome measures (PROMs) [1, 9, 10, 12, 17, 18, 27, 28, 38, 50, 59, 60].

Timing of surgery may be determined by patient or surgeon preference or access to healthcare services in some countries. In elite athletes, earlier surgery may be considered to facilitate earlier return to sport. In contrast, older or less athletic patients may trial non‐operative management first and delay surgery. A number of studies have reported that early surgery may also be associated with higher reinjury rates and complications, including arthrofibrosis [9, 25, 33, 50, 59, 60]. In contrast, delayed surgery may be associated with an increased incidence of secondary intra‐articular pathology involving the menisci and cartilage [2, 6, 17, 18, 34, 41, 42, 43, 45, 51, 52]. The optimal timing of surgery and the consequences of trialling non‐operative management are unclear.

The aim of this study was to utilize the large patient cohort of the New Zealand ACL Registry to clarify the association between the timing of ACL reconstruction and the risk of revision ACL reconstruction, concomitant meniscal and chondral injuries. It was hypothesized that a greater incidence of concomitant injuries would be observed with increasing time from injury to surgery.

## MATERIALS AND METHODS

### Ethics

Exempt from Health and Disability Ethics Committee review. All patients recorded in the New Zealand ACL Registry have signed consent forms to participate in data analysis.

### The New Zealand ACL Registry

The New Zealand ACL Registry is a nationwide registry that began in 2014 and prospectively captures data on patient, surgical and follow‐up variables. Since 2017, it has been mandatory for all orthopaedic surgeons who perform ACL reconstructions to actively participate in the registry in order to achieve recertification [37]. The operation of the registry has been declared as a protected quality assurance activity by the New Zealand Government's Ministry of Health, and all patients recorded in the registry have signed consent forms to participate. Patient demographic data are collected through a pre‐operative patient questionnaire. An intraoperative data form detailing each reconstruction procedure is completed by the surgeon. The presence of a meniscal tear and whether a repair or resection was performed was documented. The presence of chondral injury was documented by the surgeon according to the International Cartilage Repair Society (ICRS) classification.

### Patient population and inclusion criteria

Primary ACL reconstructions performed between April 2014 and December 2022 were included, allowing for a minimum follow‐up of 2 years. Patients who underwent multi‐ligament reconstruction, osteotomy, unicompartmental knee replacement, revision or contralateral ACL reconstruction prior to follow‐up were excluded.

Timing of surgery was analyzed as the time from date of ACL rupture to date of primary ACL reconstruction. Patients were categorized into five groups of surgical timing, including ≤6 weeks, 6 weeks to 3 months, 3–6 months, 6–12 months and >12 months.

### Outcomes of interest

Revision ACL reconstruction was analyzed as captured by the New Zealand ACL Registry. The capture rate of revisions by the Registry has previously been validated to be over 95% complete [44].

Concomitant medial or lateral meniscal injury was defined as a meniscal tear that was treated with either resection or repair at the time of ACL reconstruction. Concomitant chondral injury was defined as a grade 1–4 ICRS lesion in either the patellofemoral, medial or lateral compartments, as documented by the operating surgeon.

### Statistical Analyses

Descriptive statistics were provided as mean values with standard deviation (SD) or median values with interquartile ranges (IQR). Patient demographics were compared using the Chi‐square test for categorical variables and analysis of variance for continuous variables. Revision rates were compared using a univariate chi‐square test and multivariable Cox regression survival analysis with adjustment for age, gender, graft type and preinjury Marx score. The rate of concomitant injury was compared using a univariate chi‐square test and multivariable binary logistic regression with adjustment for age, gender and preinjury Marx score. Hazard ratios (HRs) and odds ratios (ORs) with 95% confidence intervals (CIs) were computed for revision risk and concomitant injuries, respectively. Patients who underwent surgery between 6 weeks and 3 months were used as the reference category when calculating risk ratios. Results were considered statistically significant at *p* < 0.05. All analyses were performed using IBM SPSS Statistics version 25.

## RESULTS

A total of 15,586 primary ACL reconstructions were analyzed (Table 1). The mean time of follow‐up was 3.8 years. With regards to surgical timing, a total of 1263 patients underwent ACL reconstruction within 6 weeks (8%), 3718 patients between 6 weeks to 3 months (24%), 5129 patients between 3 and 6 months (33%), 3223 patients between 6 and 12 months (21%) and 2253 patients more than 12 months after injury (14%).

**Table 1:** Baseline demographics.

	Time to surgery										
	≤6 weeks		6 weeks to 3 months		3–6 months		6–12 months		>12 months		
Demographic	*n*	%	*n*	%	*n*	%	*n*	%	*n*	%	*p*
*Total ACL reconstructions*	1263	8.1	3718	23.9	5129	32.9	3223	20.7	2253	14.5	
Preinjury Marx (mean)	12.6		11.8		11.6		11.0		10.5		<0.001
Age (years, mean)	27.4		28.0		28.9		30.1		32.2		<0.001
Sex											<0.001
Male	776	61.4	2133	57.4	2814	54.9	1799	55.8	1292	57.3	
Female	487	38.6	1585	42.6	2315	45.1	1424	44.2	961	42.7	
Graft type											<0.001
BTB	462	36.6	1187	31.9	1364	26.6	716	22.2	495	22.0	
Hamstring tendon	750	59.4	2327	62.6	3397	66.2	2299	71.3	1614	71.6	
Quadriceps tendon	19	1.5	108	2.9	219	4.3	130	4.0	80	3.6	
Other	32	2.5	96	2.6	149	2.9	78	2.4	64	2.8	

Abbreviations: ACL, anterior cruciate ligament; BTB, bone‐patellar tendon‐bone.

### Association between demographics and different timing of surgery

Differences in baseline demographics were noted between patients who underwent early versus delayed surgery (Table 1). As the time from injury to surgery increased, patients were more likely to report lower Marx activity scores and were older in age. The proportion of male patients and usage of bone‐patellar tendon‐bone autograft decreased as time to surgery increased.

### Association between the timing of surgery and revision rates

Delayed ACL reconstruction was associated with a reduction in the rate of revision (Table 2). Patients who underwent surgery more than 12 months after injury had the lowest rate of revision (2.4%), whereas patients who underwent surgery between 6 and 12 months had the second lowest revision rate of 3.1%. On multivariable analysis, patients in these groups had a significantly lower odds of revision (adjusted OR = 0.6 and 0.7). There was no difference in revision rates between patients who underwent ACL reconstruction within 6 weeks versus 6 weeks to 3 months versus 3–6 months.

**Table 2:** Revision ACL reconstruction (ACLR) and concomitant injuries.

	Time to surgery																	
	≤6 weeks				6 weeks to 3 months		3–6 months				6–12 months				>12 months			
Demographic	*n*	%	HR/OR (95% CI)	*p*	*n*	%	*n*	%	HR/OR (95% CI)	*p*	*n*	%	HR/OR (95% CI)	*p*	*n*	%	HR/OR (95% CI)	*p*
*Total*	1263	8.1			3718	23.9	5129	32.9			3223	20.7			2253	14.5		
Revision ACLR	61	4.8	1.1 (0.8–1.5)	0.42	174	4.7	209	4.1	0.9 (0.7–1.1)	0.34	99	3.1	0.7 (0.5–0.9)	0.01	54	2.4	0.6 (0.4–0.8)	<0.001
Meniscal resection/repair																		
Medial	475	37.6	1.1 (1.0– 1.3)	0.08	1312	35.3	1860	36.3	1.0 (0.9–1.1)	0.79	1292	40.1	1.1 (1.0–1.3)	0.01	1204	53.4	1.9 (1.7–2.2)	<0.001
Lateral	547	43.3	1.3 (1.2–1.5)	<0.001	1393	37.5	1736	33.8	0.9 (0.8–1.0)	0.003	1031	32.0	0.8 (0.7–0.9)	<0.001	756	33.6	0.9 (0.8–1.0)	0.10
Chondral injury																		
Any compartment	379	30.0	1.1 (1.0–1.3)	0.11	1060	28.5	1766	34.4	1.3 (1.2–1.4)	<0.001	1269	39.4	1.5 (1.3–1.6)	<0.001	1124	49.9	2.2 (1.9–2.4)	<0.001
Patellofemoral	154	12.2	1.1 (0.9–1.4)	0.32	448	12.0	749	14.6	1.2 (1.0–1.3)	0.03	548	17.0	1.2 (1.1–1.4)	0.01	471	20.9	1.4 (1.2–1.7)	<0.001
Medial compartment	244	19.3	1.2 (1.0–1.4)	0.09	661	17.8	1204	23.5	1.4 (1.2–1.5)	<0.001	920	28.5	1.7 (1.5–1.9)	<0.001	859	38.1	2.5 (2.2–2.8)	<0.001
Lateral compartment	100	7.9	1.0 (0.8–1.3)	0.87	301	8.1	434	8.5	1.0 (0.9–1.2)	0.77	351	10.9	1.3 (1.1–1.6)	0.00	367	16.3	2.0 (1.7–2.4)	<0.001

Abbreviations: ACL, anterior cruciate ligament; CI, confidence interval; HR, hazard ratio; OR, odds ratio.

### Association between the timing of surgery and meniscal injury

Concomitant medial meniscal injury was lowest in patients who underwent surgery between 6 weeks and 3 months after rupture (36.3%). The incidence of medial meniscal injuries was significantly greater when surgery was delayed more than 6 months after rupture. On multivariable analysis, patients who underwent surgery between 6 and 12 months after rupture had 1.1 times higher odds of medial meniscal injury, and patients who underwent surgery more than 12 months after rupture had 1.9 times higher odds (Table 2). In contrast, the incidence of lateral meniscal tears was highest in patients who underwent surgery within 6 weeks of rupture. The incidence of lateral meniscal tears progressively decreased with increasing time to surgery.

### Association between the timing of surgery and chondral injury

Patients who underwent surgery between 6 weeks and 3 months of ACL rupture had the lowest incidence of concomitant chondral injury (28.5%). For all compartments in the knee, the incidence and odds of a concomitant chondral lesion increased with increasing time from injury to surgery (Table 2). 50% of patients who underwent ACL reconstruction more than 12 months after rupture had a chondral lesion in at least one compartment.

## DISCUSSION

The most important finding of this study was that delayed surgery was associated with increasing rates of concomitant medial meniscal and chondral injuries. Patients who underwent surgery within 3 months had the lowest incidence of concomitant injuries, while surgery more than 6 months after injury resulted in the highest incidence. Earlier surgery was associated with a higher rate of revision ACL reconstruction, but was also performed in a different demographic of patients who were younger, male, more active and therefore higher‐risk.

There is a lack of consensus on the optimal timing of ACL reconstruction [15, 35, 47, 48, 54]. Recently, there have been increasing reports of an association between early surgery and a greater risk of revision surgery [9, 25, 50, 59, 60]. Although the reason for the higher risk has not been clearly identified, these studies have suggested delaying surgery to reduce the risk of repeat injury. We found that patients who underwent earlier surgery had higher revision rates, but they were also more likely to be younger, male and had higher baseline activity levels. These demographic factors have been previously identified as independent risk factors for repeat injury following ACL reconstruction [14, 26, 29, 30, 31, 32, 55, 56, 57, 58, 60]. It is possible that the association between revision risk and timing of surgery is explained by a difference in patient demographics, rather than a direct consequence of performing surgery earlier. It is important to acknowledge that observational studies, such as registry‐based studies, may offer large patient populations and statistical power, but they may be influenced by confounding factors that cannot be controlled for, and therefore, these studies cannot infer causality.

The management of ACL rupture includes non‐operative and operative interventions. The association between early surgery and patient outcomes is unclear and as a result, some clinicians may prefer a trial of non‐operative management first [19, 49]. However, the decision to delay surgery must be weighed against the potential consequences of delayed return to sport in athletes and an increased incidence of secondary intra‐articular pathology [2, 6, 17, 18, 34, 41, 42, 43, 45, 51, 52]. A recent study from the SANTI Study Group analyzed 4697 consecutive patients and found an incidence of medial meniscal injuries of 33% when surgery was performed within 3 months, 39% if between 3 and 12 months and 60% if more than 12 months after rupture [20]. The authors also found that a longer time to surgery was associated with more complex meniscal tears that may be more difficult to repair. This supports the findings of Tashiro et al., who suggested that meniscal ramp lesions were associated with greater delay to surgery [53]. Biomechanical studies have demonstrated the effect of meniscal ramp lesions on anterior and rotational laxity [11, 23]. Unfortunately, detecting ramp lesions may be difficult, and failure to do so may lead to progression into full lesions over time, especially with delayed surgery [5, 24].

Erard et al. and Cance et al. analyzed 1840 patients undergoing ACL reconstruction and found that a delay of more than 12 months was associated with a higher incidence of medial meniscal tears and chondral injury, but not a delay of 3 or 6 months [4, 13]. In our study, the lowest incidence of medial meniscal tears was observed in patients who underwent surgery between 6 weeks and 3 months after rupture; however, the odds of concomitant injury significantly increased when surgery was performed more than 6 months after injury. In addition, the incidence of chondral injury progressively increased for every delay of 3 months. Our findings are identical to two meta‐analyses performed by Prodromidis et al. that found that surgery within 3 months was associated with the lowest incidence of secondary intra‐articular pathology [42, 43].

An interesting finding of the study was a higher rate of lateral meniscal injury in patients undergoing early surgery. The contrasting incidence between medial and lateral meniscal tears may be explained by differences in the function of the menisci and the mechanism of injury. The medial meniscus functions as a secondary knee stabilizer in anterior tibial translation; therefore, injury may be more common with chronic ACL insufficiency [8, 36, 40]. In contrast, lateral meniscal tears, particularly bucket‐handle tears, frequently occur in acute traumatic knee injuries, may be more symptomatic for the patient and require semi‐urgent treatment [7, 16, 22, 39]. The greater incidence of lateral meniscal tears observed in patients undergoing earlier surgery may therefore be explained as a consequence of trauma. In contrast, the greater incidence of medial meniscal tears and chondral injuries observed in patients undergoing delayed surgery may be a consequence of delaying surgery in an ACL‐deficient knee [3]. The ACL plays a crucial role in both anteroposterior and rotational stability of the knee. Therefore, in the ACL‐deficient knee, increased forces may be transferred to the medial meniscus and chondral surfaces resulting in concomitant injury over time, but also through further episodes of knee instability. The findings of this study therefore support performing earlier ACL reconstruction to reduce the odds of concomitant injuries.

This study had a number of limitations. Although registry studies provide large patient populations and excellent statistical power, they are limited to identifying associations and cannot infer causality. In addition, there was a lack of data on the type and complexity of each meniscal tear. A randomized controlled trial analyzing the incidence of concomitant injuries with different timings of surgery would be able to address these limitations but may be expensive and difficult to adequately power. Another limitation is that the exact timing of meniscal or chondral injury could not be determined and therefore it could not be identified whether the injury was sustained at the time of index ACL injury.

## CONCLUSION

Delayed ACL reconstruction is associated with a greater incidence of concomitant medial meniscal and chondral injury and should be considered when trialling non‐operative management for ACL rupture.

## AUTHOR CONTRIBUTIONS

**Richard Rahardja**: Conceptualization; data curation; formal analysis; investigation; methodology; validation; visualization; writing. **Hamish Love**: Data curation; project administration; resources; writing. **Mark G. Clatworthy**: Data curation; project administration; resources; writing. **Simon W. Young**: Data curation; methodology; project administration; supervision; validation; writing.

## CONFLICT OF INTEREST STATEMENT

The authors declare no conflicts of interest.

## ETHICS STATEMENT

Exempt from Health and Disability Ethics Committee review as an audit activity. All patients had signed a consent form to be recorded in the Registry for the purpose of research.

## Data Availability

Data available on request from the authors.
